# 112.5 Gbit/s long reach passive optical network with over 31 dB power budget enabled by semiconductor optical amplifiers

**DOI:** 10.1038/s41598-025-12857-z

**Published:** 2025-07-29

**Authors:** Ahmed Galib Reza, Lakshmi Narayanan Venkatasubramani, Liam P. Barry

**Affiliations:** School of Electronic Engineering, Dublin City University, Glasnevin, Dublin 9, Ireland

**Keywords:** Intensity modulation and direct detection, Passive optical networks, Pulse amplitude modulation, Semiconductor optical amplifier, Probabilistic shaping, Optics and photonics, Electrical and electronic engineering

## Abstract

We experimentally demonstrate the downstream transmission of 112.5 Gbit/s pulse amplitude modulated (PAM) signals in the O-band for future time-division multiplexed long-reach passive optical networks (LR-PONs). For the first time, this work demonstrates the use of a commercial quantum-well semiconductor optical amplifier (QW-SOA) in the remote node (RN) of a 100G-class PON to extend the transmission distance between the optical line terminal and the optical network unit. Besides, we discuss the performance of a QW-SOA in amplifying uniform and probabilistically shaped (PS) continuous-mode downstream PAM signals, such as PAM-4, PAM-8, and PS-PAM-8, and also discuss its potential during upstream burst-mode operations. Considering the hard-decision low-density parity-check bit error ratio limit of 1 × 10^− 2^, the optical power budgets of 32.85 dB and 31.3 dB are achieved for the downstream PAM-4 and PS-PAM-8 signals, respectively, using a truncated T-spaced Volterra nonlinear equalizer after 112.5 Gbit/s SOA-amplified transmissions over a 50 km single-mode fiber, satisfying the class N2 power budget requirement of 31 dB for PONs. To the best of our knowledge, this is the highest power budget ever reported for 100G-class LR-PON with 50 km optical reach employing a commercial QW-SOA in the RN of a PON.

## Introduction

The passive optical network (PON) is a key enabling technology that cost-effectively provides high-speed broadband access services to end-users. Due to the rapid proliferation of state-of-the-art disruptive technologies and services, such as social networking, the Internet of Things (IoT), artificial intelligence (AI), 4 K/8K video streaming, etc., the current PON technology and architecture are now confronting new challenges, as they look to support the unprecedented growing demand for capacity with low latency and cost^1,2^.

The new 25G PON is currently under deployment, which utilizes the standard time-division multiplexing (TDM) technology to deliver 25 Gbit/s symmetrical bit rates over O-band, adhering to class N1 power budget requirements of 29 dB through a high launch power^3^. The 50G PON or higher-speed PON (HSP), expected to be commercially available by 2025, will still use pulse amplitude modulation-2 (PAM-2), high launch power, and intensity-modulation and direct-detection (IM/DD) techniques to reduce the overall system cost and complexity^4–6^. Meanwhile, the standardization bodies such as the International Telecommunication Union-Telecommunication Standardization Sector (ITU-T) and the Institute of Electrical and Electronics Engineers (IEEE) are now exploring the options for a cost-effective 100G PON or very high-speed PON (VHSP) capable to coexist with the legacy PON and to provide more bandwidth and speed to provision the next-generation emerging technologies and services^6,7^. As the PON market is very cost-sensitive, higher-order modulation formats, such as PAM-4 and PAM-8, can be a suitable option to boost the overall system capacity of an IM/DD PON without requiring high-bandwidth components^8^ or moving to coherent transmission. For high-speed PONs, the 1310-nm transmission window is a better option since the dispersion is nearly zero at the O-band. Although there is a relatively higher attenuation factor compared with the C-band, this can be alleviated through higher launch power into the fiber and/or optical amplification using semiconductor optical amplifiers (SOAs), which have broad gain bandwidth, compact size, low cost, and potential for monolithic or hybrid integration^9^. It is well known that systems employing high launch power and SOA amplification are typically governed by fiber and SOA-induced nonlinearities, respectively, such as self-phase modulation, stimulated Brillouin scattering (SBS), stimulated Raman scattering (SRS), polarization sensitivity, inter-channel crosstalk, and gain saturation-induced pattern effects. Besides, multi-level signals are more susceptible to linear and nonlinear signal distortions and require a higher signal-to-noise ratio (SNR). However, recent advancements in digital signal processing (DSP) techniques can now allow the mitigation of such distortions and increase the capacity and coverage area of an IM/DD PON. Various types of DSP techniques are widely studied and found effective^10–13^, such as feed-forward equalizer (FFE), Volterra nonlinear equalizer (VNLE), decision feedback equalizer (DFE), machine learning (ML), etc. Another DSP technique, called probabilistic shaping (PS)^14^, has demonstrated strong resilience against nonlinear effects and has been considered recently for the adoption of spectrally efficient modulation formats, such as PAM-8, PAM-16, etc^15,16^.

In this regard, the 100G PON may be based on PAM-4 in the O-band, high launch power into the fiber, DSP, and optical amplifiers to support the class N1 loss budget^6^. Recently, there has been growing attention to a new PON architecture called long-reach PON (LR-PON), which extends the typical 20 km optical reach of the classic PON up to 100 km. The main benefits of LR-PON include combined metro and core access networks, wider coverage area, consolidated central office (CO) that could decrease the total number of COs in the network, reduced capital and operational expenditures (CAPEX/OPEX), etc^17,18^. Through the adoption of coherent technology, a high-speed LR-PON with large splitting ratios can be realized^19,20^; however, this will increase the cost, complexity, and power consumption of standard PONs beyond the current acceptable levels. The plausible target of the next-generation LR-PON might be to support at least 100 Gbit/s net data rates with a class N1 power budget utilizing IM/DD technology.

Several multiplexing techniques have been proposed to realize high-speed LR-PONs, such as TDM^21–24^, orthogonal frequency-division multiplexing (OFDM)^25,26^, and wavelength-division multiplexing (WDM)^27^. For instance, 100 Gbit/s/λ linear and nonlinear DSP-enabled PAM-4 signal transmission is reported over 50 km with a power budget of 29.3 dB using a directly modulated laser (DML) and an SOA^21^; 100 Gbit/s PAM-4 transmissions are demonstrated over 100 km with a power budget of 29 dB in the C-band using an IQ Mach–Zehnder modulator (MZM) for dispersion pre-compensation and nonlinear DSP techniques^23^; and transmission of 50 Gbit/s OFDM signal is demonstrated using an EDFA with a loss budget of 28 dB^26^. TDM and OFDM are the simplest multiplexing techniques in terms of wavelength management. However, they both broadcast high-speed multiplexed data on a single wavelength in the downstream direction, and each optical network unit (ONU) is required to process the entire data stream to transmit/receive a fraction of the aggregated data. Unlike TDM- and OFDM-PON, the ONU can be equipped with a low-bandwidth transceiver in a WDM-PON, as it is not required to process all the multiplexed data. However, the WDM-PON incurs very significant passive optical losses, and it might be challenging to realize an LR-PON utilizing the WDM technique that could support a link loss budget with a large splitting ratio. Besides, the ITU-T has chosen TDM technology for 50G PON^4^, which provides a data rate of 50 Gbit/s over a single channel. In this regard, the next-generation 100G LR-PON could target 100 Gbit/s upstream and downstream speeds on a single wavelength over a long distance, incorporating TDM technology.

In this paper, we propose a new architectural design concept for LR-PONs utilizing the low dispersion transmission window of 1310 nm for long-reach data transmission and overcoming high transmission losses with the help of an SOA in the remote node (RN) of a PON, which potentially could also increase the number of supported users in the network. Optically amplified RNs were proposed for 10G PONs to overcome the transmission and passive losses in the network^28–30^. An SOA in the RN could be implemented for burst-mode optical amplification^28^ or as a PON extender box^29,30^ that could potentially decrease the cost and power consumption of the PON. Recently, 2 × 53 Gbit/s DWDM PAM-4 transmissions were demonstrated using an SOA-amplified RN^31^. The PON imposes a stringent power budget requirement, which is critically challenging for IM/DD systems operating at a data rate of 100 Gbit/s or beyond. Besides, the performance of an SOA-amplified high-speed PON carrying multi-level signaling is severely impacted by the SOA-induced amplified spontaneous emission (ASE) noise and nonlinearities, such as patterning effects^32^. For the first time, by utilizing high launch power and SOA-amplified RN, we experimentally demonstrate the downstream transmission of single-lane 112.5 Gbit/s PAM signals over 50 km of a single-mode fiber (SMF) in the O-band for the next-generation 100G-class LR TDM-PONs. Considering the bandwidth limitations and SOA-induced nonlinearities, we investigate the possible implementations of higher-order modulation formats, such as PAM-4, PAM-8, and PS-PAM-8 in an SOA-amplified LR-PON, and low-complexity DSP techniques, such as T-spaced FFE and truncated second-order VNLE, to mitigate the linear and nonlinear signal distortions. After VNLE, both PAM-4 and PS-PAM-8 could achieve a power budget of 32.85 dB and 31.3 dB, respectively, at a bit error ratio (BER) threshold of 1 × 10^− 2^, satisfying the class N2 power budget requirements of 31 dB defined in NG-PON2^33^. To the best of our knowledge, this is the highest power budget ever achieved for commercial quantum-well (QW) SOA-amplified 100G-class LR-PON with an optical reach distance of 50 km.

**Fig. 1:**
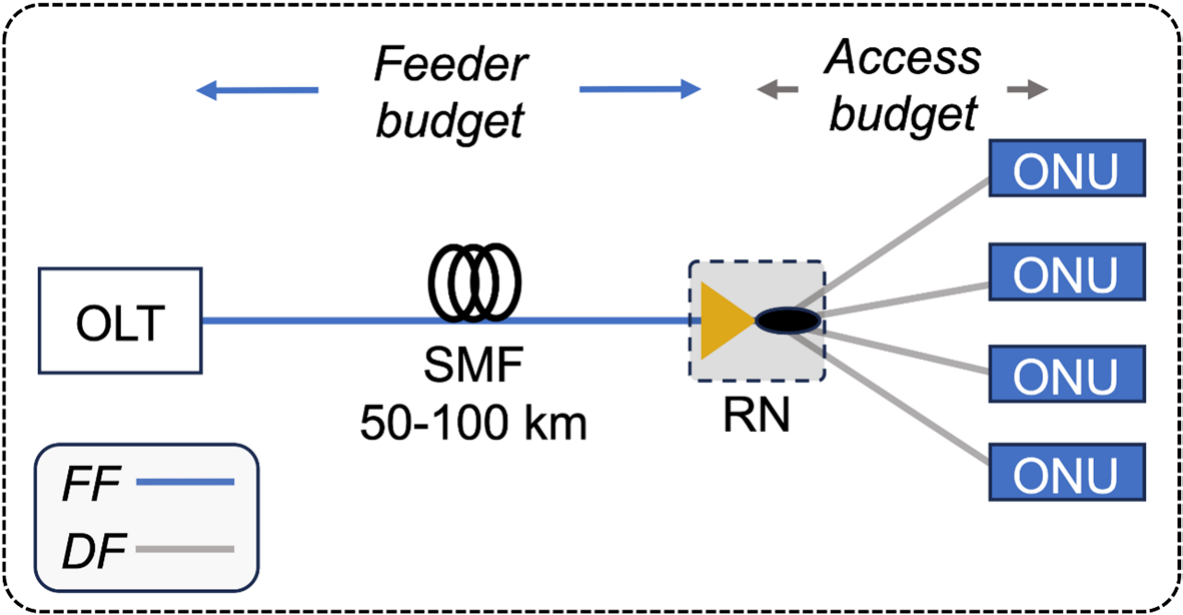
Conceptual schematic diagram for SOA-amplified LR-PON.

## Next-generation long-reach PON

A conceptual design for the next-generation LR-PON is presented in Fig. 1. The proposed architecture utilizes the optical signal amplification mechanism by placing an SOA in the RN of the next-generation LR-PON to overcome the transmission loss over a 50–100 km long feeder fiber (FF) in the O-band and enable broadcasting of the TDM signals with sufficient optical power levels required for reliable 100G transmissions to all the connected users in the network via distribution fibers (DFs)^31^. A passive splitter is usually placed in the network to broadcast optical signals in a PON. The use of an SOA in the RN could increase the total loss budget of the PON, which can be calculated by combining the feeder (optical loss incurred from the optical line terminal (OLT) to RN) and access budgets (optical power loss from the RN to the ONU). To the best of our knowledge, the performance of such an SOA-amplified LR-PON has never been investigated before and calls for attention as several major factors that might limit the performance, such as fiber and SOA-induced nonlinearities, signal distortions due to bandwidth constraints, ASE noise, photodetector (PD) sensitivity, etc.

**Fig. 2:**
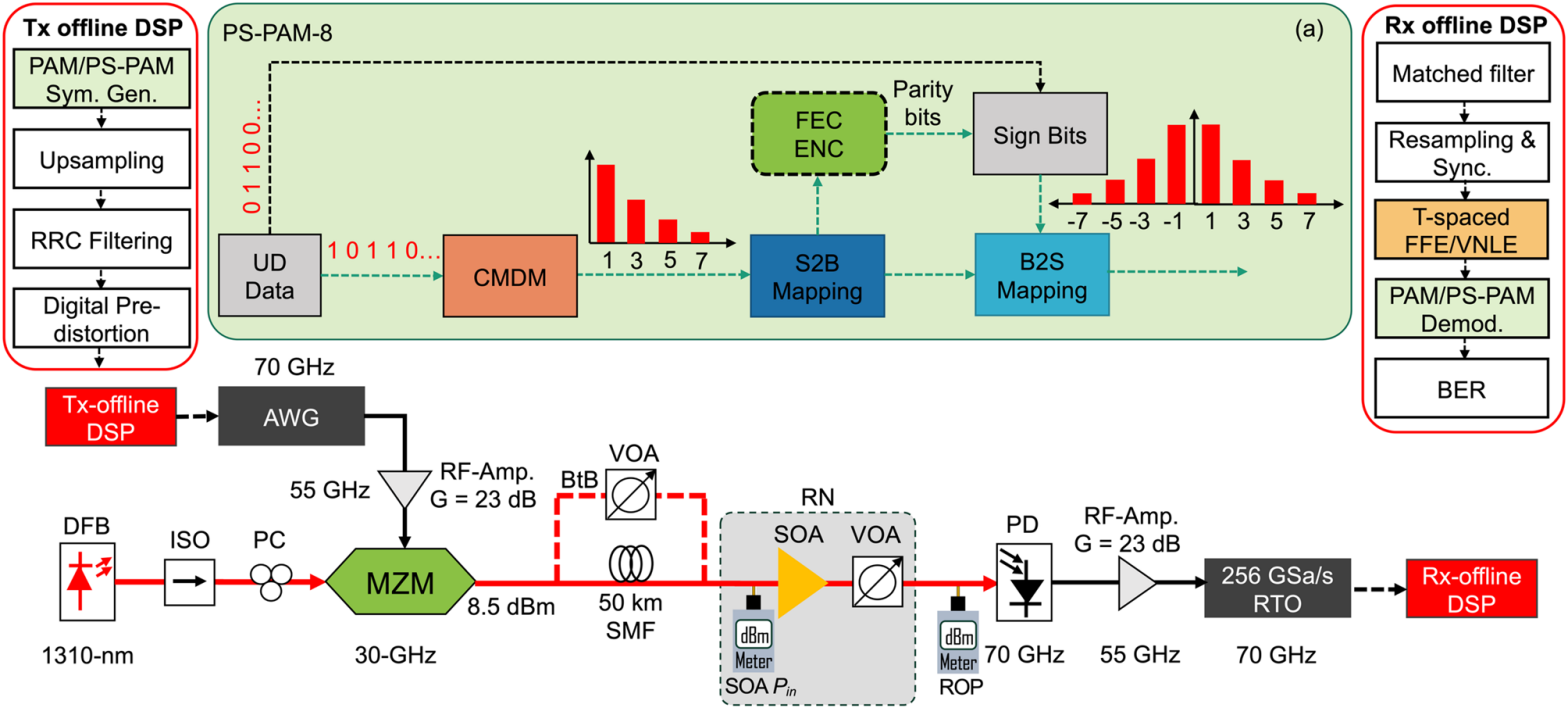
Experimental setup for the next-generation ≈ 112.5 Gbit/s LR-PON.

The experimental setup for LR-PON is illustrated in Fig. 2. To realize the next-generation LR-PON, a commercial QW-SOA amplified ≈ 112.5 Gbit/s PAM-4, PAM-8, and PS-PAM-8 signal transmissions are demonstrated in the O-band over 50 km SMF. At the transmitter-side offline DSP block, the gray-coded PAM-4 and PAM-8 symbols are generated in MATLAB with a sequence length of 2^16^ symbols that follow a uniform distribution. For the generation of PS-PAM-8 symbols (Fig. 2(a)), the uniformly distributed binary data is split into two parts: the first two-thirds of the total binary data is mapped to Maxwell-Boltzmann (MB)-distributed unsigned PAM-8 levels according to a predefined entropy using a lookup table based on a cost-minimizing distribution matcher (CMDM) algorithm^15^. After binary labeling using Binary Reflected Gray Coding (BRGC), the remaining one-third of the binary data is affixed to the signed bits of the binary labeling. Finally, the PS-PAM-8 is generated after bit-to-symbol (B2S) mapping^15,16^. It is worth mentioning that a portion of the uniformly distributed binary sign data can be replaced with forward error-correcting (FEC) parity bits without altering the probability distribution of the PS-PAM-8 symbols.

To maintain a comparable data rate of ≈ 112.5 Gbit/s, the symbol rates for PAM-4, PAM-8, and PS-PAM-8 modulations are set at 56.25 GBaud, 37.5 GBaud, and 42 GBaud, respectively. The digital PAM-4, PAM-8, and PS-PAM-8 symbols are up-sampled to 4, 6, and 5 times, respectively, and pulse-shaped using a root-raised cosine (RRC) filter with a roll-off factor of 0.1. To reduce the influence of modulator nonlinearities, the *arcsin* pre-distortion technique is applied to the digital data so that the PAM symbol levels become equidistant upon modulation. The digital PAM signals are now loaded to an arbitrary waveform generator (AWG), running at 225 GSa/s for PAM-4 and PAM-8 signals or at 210 GSa/s for PS-PAM-8 signals. The analog bandwidth of the AWG is 70 GHz. In general, a larger peak-to-peak voltage (Vpp) swing is required as the modulation order grows, leading to higher nonlinear signal distortions and BERs, if not set properly. The Vpp of the AWG output is optimized at 260 mV for PAM-4 and PS-PAM-8 signals and 300 mV for PAM-8 signals, which is amplified using an RF amplifier with a bandwidth of 55 GHz. The RF amplifier provides a gain of 23 dB, and the 3 dB gain compression (*P*_3dB_) appears at 19 dBm. The amplified electrical signal drives a commercial lithium niobate (LiNbO_3_)-based MZM, which is biased at the quadrature point. The electro-optic (E-O) S21 response curve of the MZM shows that it can offer a 3-dB modulation bandwidth of 30 GHz, which can range up to a 6-dB bandwidth of 40 GHz due to its slow roll-off factor. In the experiment, a high-power distributed feedback (DFB) laser serves as an optical carrier, which emits light at 1310 nm with an optical output power of 16 dBm for a bias current of 325 mA. Upon modulation, the ≈ 112.5 Gbit/s PAM signal with an optical power of 8.5 dBm is directly launched into an SMF for back-to-back (BtB) system characterization and transmissions over a distance of 50 km.

After transmission over a 50 km feeder fiber, the received signal is amplified with an SOA placed in the RN to compensate for transmission loss and to improve the power budget. For the BtB transmission case, a variable optical attenuator (VOA) is placed in the system before the SOA to vary the input power into the SOA. To emulate the split loss of a typical TDM-PON system and to adjust the received optical power (ROP), the optically amplified signal is attenuated by a VOA before being directly detected by a 70 GHz bandwidth PD. As shown in Fig. 2, the ROP is always measured at the input of the PD. After detection, the signal is amplified by a 55 GHz RF amplifier with a gain of 23 dB and captured using a real-time oscilloscope (RTO) running at 256 GSa/s for signal recovery using offline signal processing. At the receiver-side DSP, the captured signals are matched filtered using an RRC filter, resampled to 1 sample/symbol, time aligned, and equalized individually using a 32-tap FFE and a truncated VNLE for comparison, which considers 32 linear taps and ten nonlinear taps with the beating of up to three neighbors for residual nonlinearity compensation^8,11^. After equalization, the signal is demodulated, and the BER performance is measured through counting.

## Experimental results

**Fig. 3:**
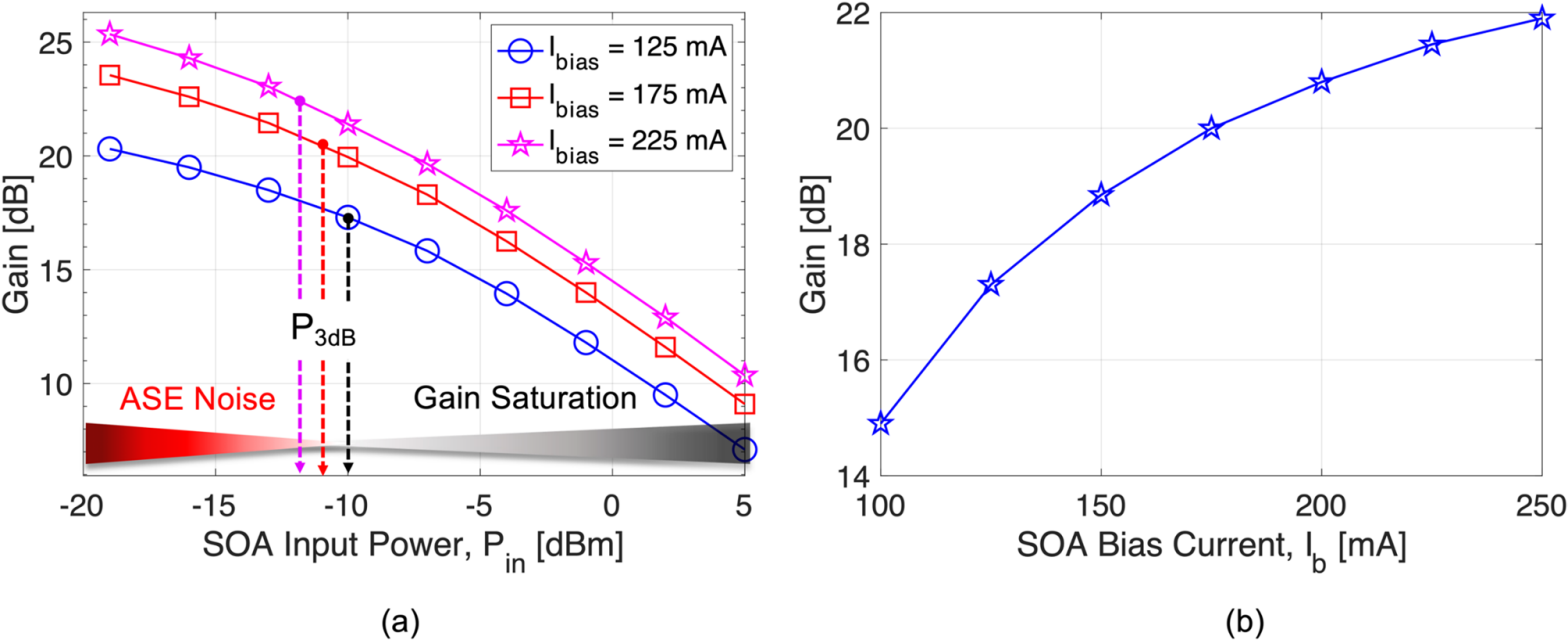
(**a**) SOA gain as a function of SOA input signal power for various bias currents. (**b**) SOA gain versus bias current for an input signal power of -10 dBm.

Figure 3(a) presents the gain curve of a commercial InP/InGaAsP multiple quantum well (MQW) layer SOA as a function of input power (*P*_in_) into the SOA for three different bias currents of 125 mA, 175 mA, and 225 mA. Regardless of the bias current, we can see that the SOA gain continues to fall as input power increases. We find that the SOA input signal power at a 3dB compression point, at which the SOA gain is reduced by 3 dB from the unsaturated point, can vary between − 12 dBm and − 10 dBm, depending on the SOA bias current. If we move towards the right from the *P*_3dB_ point, the SOA input signal goes above *P*_3dB_ (*P*_in_ > *P*_3dB_), which could potentially saturate the SOA. In the gain saturation regime, the SOA exhibits patterning effects due to a carrier lifetime in the active region of the SOA. At the low input power (*P*_in_ = -19 dBm < < *P*_3dB_), the SOA enters the unsaturated regime and can provide larger gains of about 20.3 dB, 23.5 dB, and 25.3 dB for the bias currents of 125 mA, 175 mA, and 225 mA, respectively. In this regime, the SOA exhibits a higher noise figure (NF) as the spontaneous-spontaneous beat noise prevails during the amplification process. Thus, the SOA-induced ASE noise primarily dominates the signal quality.

Figure 3(b) shows the gains of the SOA as a function of the bias current. In the figure, the SOA input signal power is fixed at -10 dBm. It can be found that the SOA gain can be increased by simply setting a higher bias current. However, the SOA gain curve indicates gain saturation at a higher SOA bias current, which could result in gain saturation-induced nonlinear signal distortions during the amplification process. Considering the trade-off between the SOA gain and nonlinear signal distortions, finding a suitable SOA bias current for amplifying high-speed multi-level signals is critical.

**Fig. 4:**
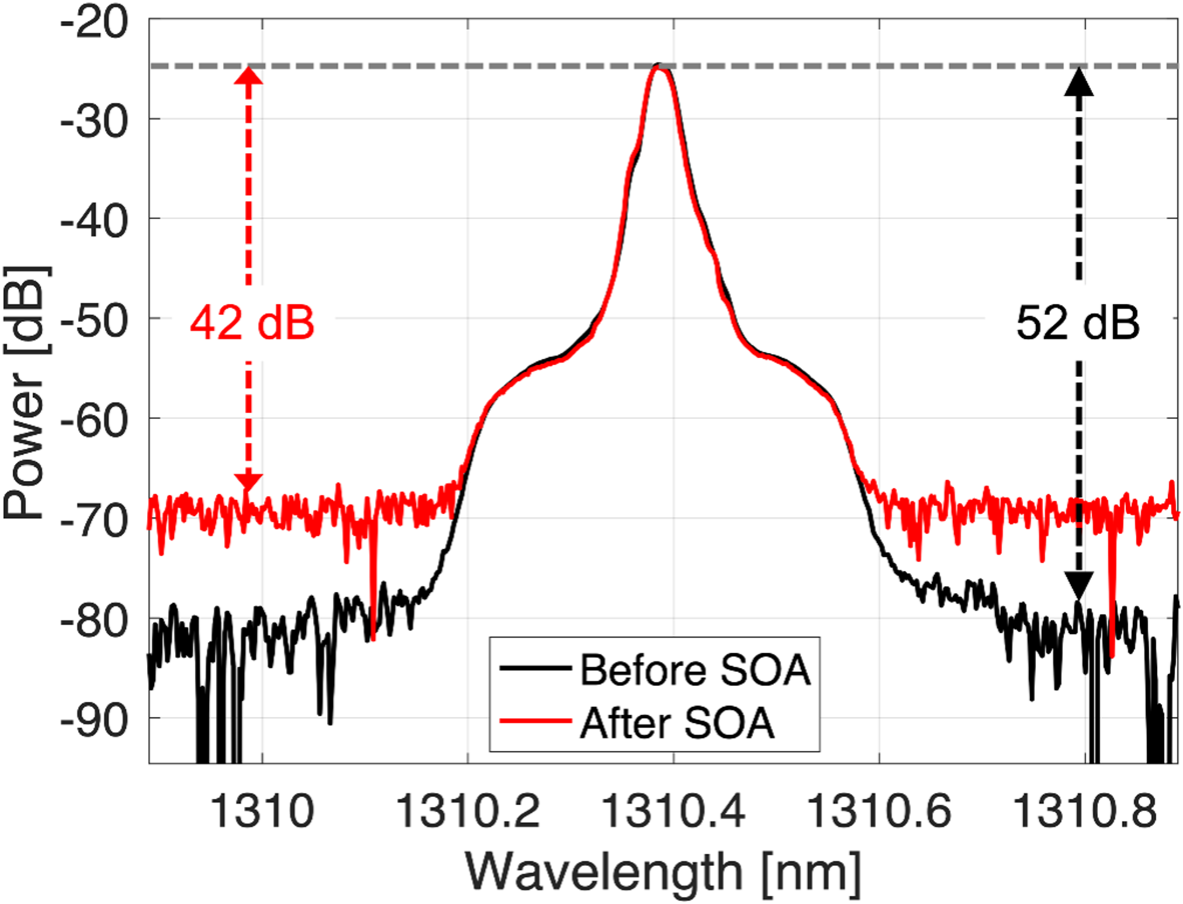
Modulated optical spectra (measured at 0.01 nm) of 56.25 GBaud PAM-4 signal before and after amplification with an SOA.

The optical spectra of 56.25 GBaud PAM-4 signals measured at 0.01 nm resolution bandwidth before and after optical amplifications using an SOA are shown in Fig. 4. In the figure, the input signal power of -10 dBm and a constant bias current of 125 mA are supplied to the SOA. With a VOA, the same power levels before and after the SOA (which provides ~ 17.3 dB gain in this case) are maintained in the plot, enabling a head-to-head comparison between the optical signal-to-noise ratio (OSNR) before and after the SOA. We can see that after SOA amplification, the OSNR can be degraded by about 10 dB. However, the measured OSNR of the amplified signal can still be as high as 42 dB, encouraging the transmission of higher-order modulation formats such as PAM-4 or PAM-8 at high baud rates. In addition, the SOA offers a 3-dB bandwidth of about 70 nm for a center wavelength of 1310 nm.

**Fig. 5:**
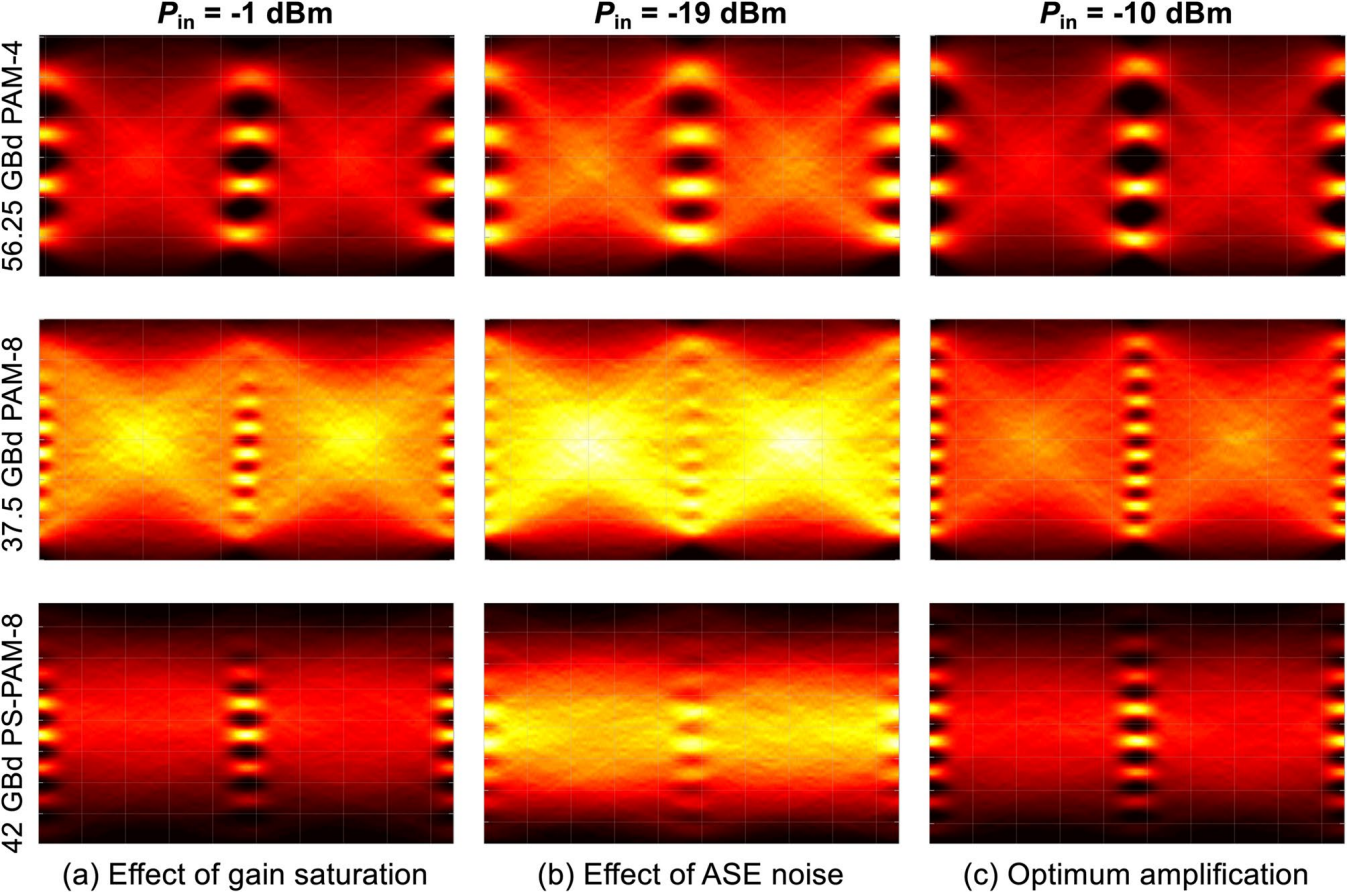
Measured eye diagrams of ≈ 112.5 Gbit/s PAM-4, PAM-8, and PS-PAM-8 signals at an ROP of 0 dBm after BtB amplified transmissions using an SOA for input powers of (**a**) -1 dBm, (**b**) -19 dBm, and (**c**) -10 dBm.

Figure 5 shows the measured eye diagrams of 56.25 GBaud PAM-4, 37.5 GBaud PAM-8, and 42 GBaud PS-PAM-8 signals at an ROP of 0 dBm in a BtB system for three different input powers into the SOA. Throughout the experiment, the entropy of the PS-PAM-8 signals is held constant at 2.675 bits/symbol so that the total achievable data rates for all modulation formats are comparable. To mitigate the bandwidth limitations of the system, a 32-tap FFE is applied at the receiver side, which operates at the sampling baud rate. In Fig. 5(a), for an SOA input signal power of -1 dBm, a significant gain saturation can be observed, particularly for PAM-8 signals, as the sixth to eighth amplitude levels of the PAM-8 signals are completely squashed and indistinguishable. The PS-PAM-8 eye diagram suggests that the PS can exhibit strong resilience against SOA-induced pattern effects and nonlinear compressions in the higher energy PAM levels. Decreasing the number of levels in the input signal, such as through the use of PAM-4, can reduce the impact of gain saturation effects at the expense of reduced spectral efficiency, which imposes the requirements for higher bandwidth optical/electrical components, and high-speed digital-to-analog/analog-to-digital converters (DACs/ADCs). When the SOA input signal power is -19 dBm (Fig. 5(b)), the SOA is operated in the unsaturated region, and the ASE noise generated by the SOA governs the system performance across all modulation formats. The eye diagram confirms that the higher-order modulation formats demand higher SNR to have the same level of performance. In contrast with PAM-8, the PS-PAM-8 offers marginally better eye openings, meaning that the efficacy of PS in alleviating SOA-induced ASE noise is somewhat limited. Compared with the two other SOA input powers, at -10 dBm, the best eye openings are observed for all the modulation formats considered in this paper, implying that optimizing the input power and bias current is critically important to obtain the best system performance with an extended optical reach.

**Fig. 6:**
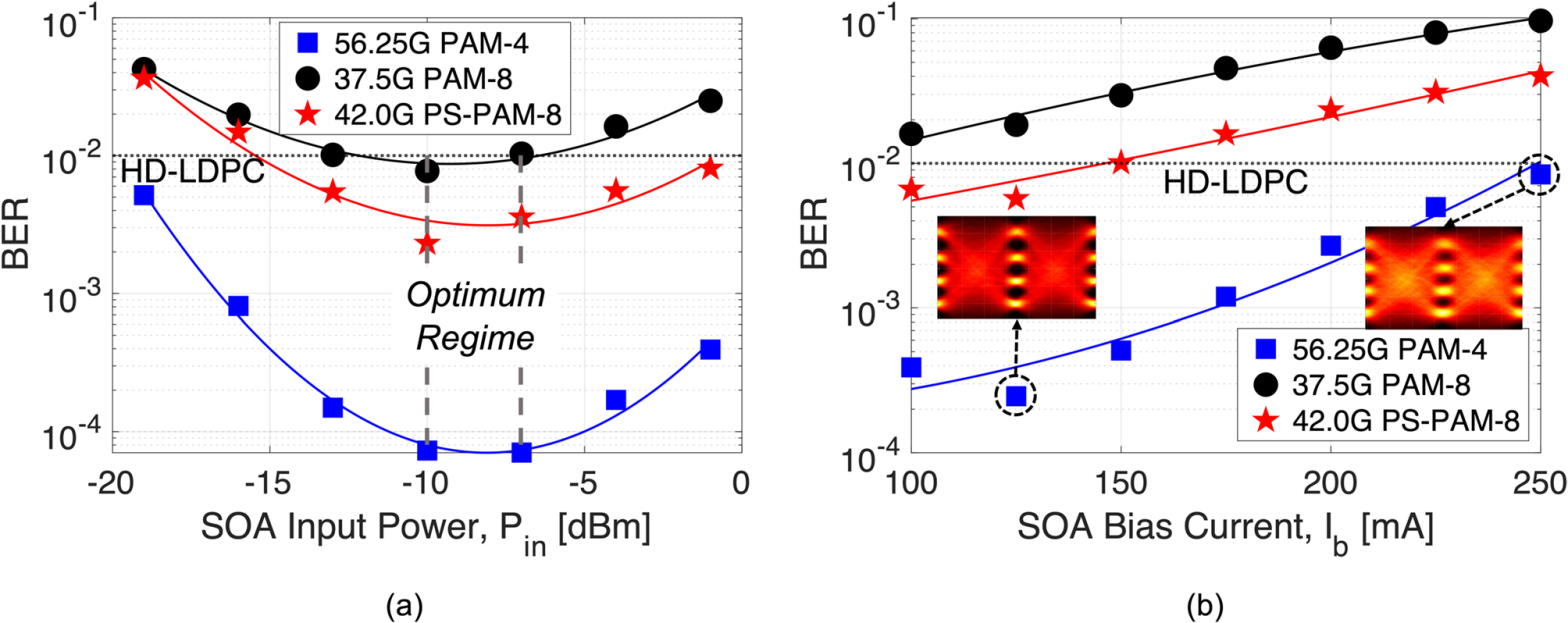
Investigation of the SOA gain dynamics and BER performance as a consequence of the change of input signal power and bias current. In the plot, the square, circle, and star pentagon markers show the performances of ≈ 112.5 Gbit/s PAM-4, PAM-8, and PS-PAM-8 signals, respectively. (**a**) BER versus SOA input powers for the fixed bias current at 125 mA after BtB signal transmissions at an ROP of 0 dBm. (**b**) BER versus SOA bias currents after 50 km at an ROP of 0 dBm for the SOA input power fixed at -10 dBm.

Next, we experimentally examine the input power dynamic range of the SOA on system performance. In Fig. 6(a), we plot the BER results of ≈ 112.5 Gbit/s PAM-4, PAM-8, and PS-PAM-8 signals as a function of SOA input power after equalizations with an FFE for the BtB transmission cases. A constant bias current of 125 mA is applied to the SOA, and the ROP is fixed to 0 dBm in all cases. In the plot, the equalized BER performances of PAM-4, PAM-8, and PS-PAM-8 signals are denoted by the square, circle, and star pentagon markers, respectively. We can see that the performance of PAM-8 signals is the poorest among the three modulation formats for any SOA input signal power, and it can barely touch the pre-FEC hard-decision low-density parity-check (HD-LDPC) BER limit of 1 × 10^− 2^ for SOA input power ranging only from − 7 dBm to -13 dBm, which supports our findings presented in Fig. 5 that the PAM-8 signals are more susceptible to linear and nonlinear distortions and have stringent SNR requirements. Unlike PAM-8, the PS-PAM-8 shows robustness against such impairments due to the SOA, as it can reach the HD-LDPC level BER performance quite comfortably across a wide range of SOA input powers (-1 dBm to -15 dBm), which is important for implementation in practical PON scenarios. The BER performance of PAM-4 indicates that the distortions caused by the SOA only trigger serious concern for the four-level signals when amplified with an overly saturated and unsaturated SOA. It can be seen that all multi-level modulation formats suffer from nonlinearity and level-dependent noise, and the equalized BER trend lines indicate a deterioration in the BER performances due to the gain saturation effects and ASE noise in the saturated and unsaturated regions of the SOA, respectively. However, the best BER performances for all modulation formats can only be achieved when the SOA input power falls in the optimum regime (-7 dBm to -10 dBm), as this regime provides an optimal balance point assessing the gain saturation and ASE noise penalties. Therefore, it can be intuitively concluded that the best way to minimize the degradation in the BER performance is to optimize the input power into the SOA and/or implement more advanced signal processing technologies. Even though this work focuses on the downstream operation, the results presented above indicate that the SOA dynamic range is well above 18 dB for PAM-4 signals at the HD-LDPC BER limit, making it potentially suitable for burst-mode operation. Nonetheless, the SOA dynamic range is about 3.5 dB narrower (about 14.5 dB) for the PS-PAM-8 signals compared with the PAM-4 signals. A wider SOA dynamic range is important to realize bursty upstream data transmission, as the optical signal power levels are different from burst-to-burst due to various path lengths between the OLT and ONUs. In the proposed LR-PON design, the amplified upstream data transmission can be realized using a shared or dedicated SOA in the RN. In a shared SOA configuration, a single SOA located in the RN amplifies upstream and downstream data simultaneously, leading to nonlinear signal distortions due to cross-gain modulation (XGM) that causes interferences between the downstream and upstream signals, which can become more severe as the burst mode upstream signal turns on and off, however the feasibility of this implementation has been presented in^34^. On the other hand, for a dedicated SOA configuration, two separate SOAs are utilized in the RN with the help of two three-port optical circulators, one in each direction. In such a configuration, the main distortions will be the added ASE and patterning effects caused by the gain saturation of the SOA, as discussed in this paper^32^.

To evaluate the impact of SOA bias current on the system transmission performance, Fig. 6(b) presents the 32-tap FFE equalized BER results of all modulation formats for several SOA bias currents with an SOA input power of -10 dBm and at an ROP of 0 dBm after 50 km transmissions. It can be observed that the BER of all modulation formats degrades with an increase in the bias current. The PAM-4 eye diagrams, given in the insets of Fig. 6(b) for the bias currents of 125 mA and 250 mA, indicate higher ASE noise and nonlinear signal distortions at higher bias currents. It can be seen that the uniform PAM-8 signals cannot achieve the BERs below the HD-LDPC BER limit for any bias currents. On the other hand, PS-PAM-8 can meet the target BER level of 1 × 10^− 2^ when the SOA bias current is set to 150 mA or less. At a bias current of 125 mA, the SOA provides an adequate gain of 17.3 dB while amplifying higher-order PAM signals in the RN or local exchange of an optical access network, which would be chosen for the subsequent experiments in this paper.

**Fig. 7:**
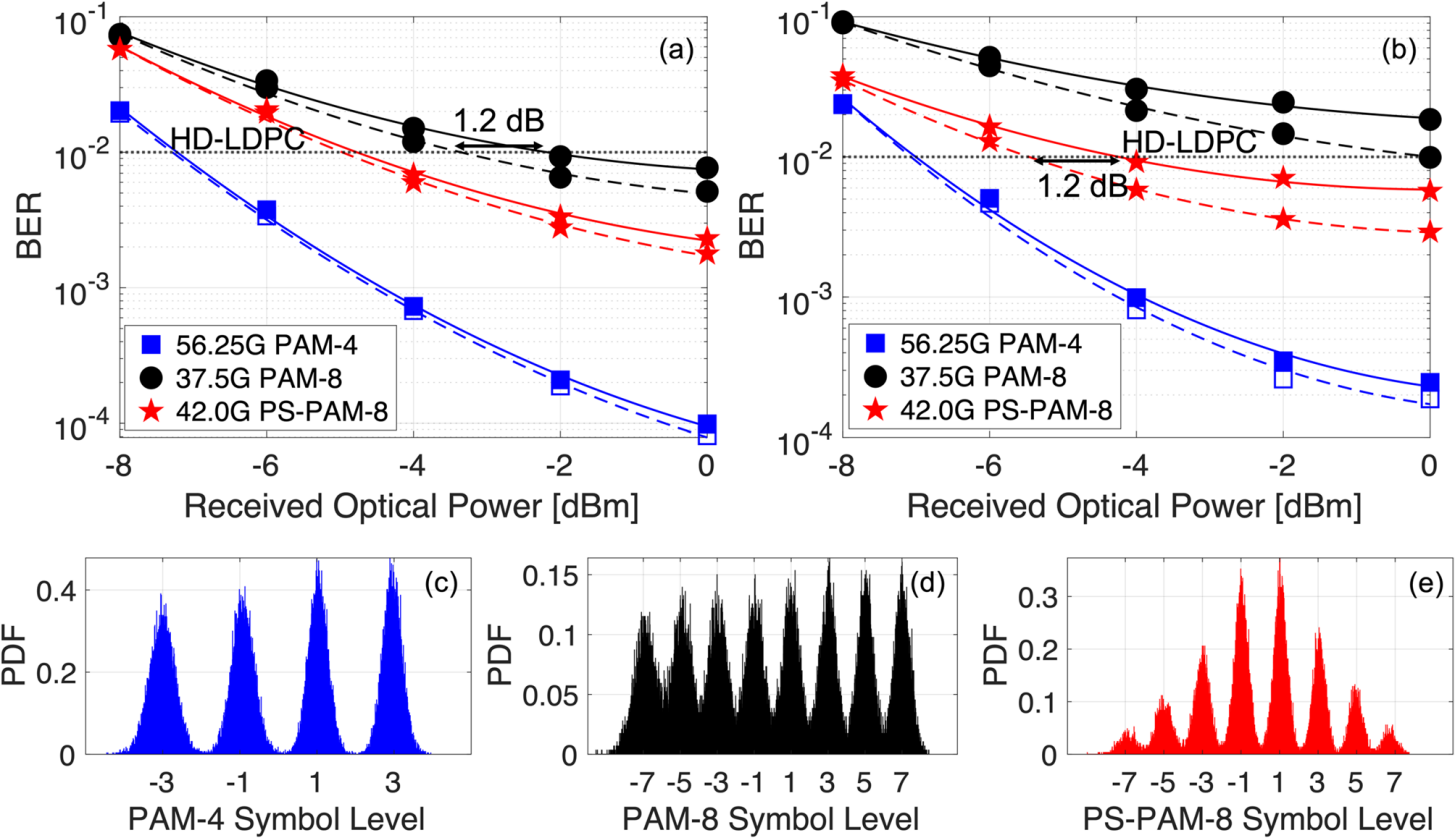
Experimental results for optically amplified 56.25 GBaud PAM-4, 37.5 GBaud PAM-8, and 42 GBaud PS-PAM-8 signal transmissions. The square, circle, and star pentagon markers denote the BERs of PAM-4, PAM-8, and PS-PAM-8, respectively. BERs versus ROPs after transmissions over (**a**) BtB, and (**b**) 50 km SMF links. The histograms of amplitude distributions at an ROP = 0 dBm for (**c**) PAM-4, (**d**) PAM-8, and (**e**) PS-PAM-8 symbol levels.

In Fig. 7(a), we present the BER performances of ≈ 112.5 Gbit/s PAM signals as a function of ROP in the case of a BtB transmission link. In the experiment, the SOA bias current and input signal power are held constant at 125 mA and − 10 dBm, respectively. For comparison, both FFE and VNLE are applied in this case for symbol equalizations, which are indicated by the solid and dashed lines, respectively. It is worth mentioning that the introduction of VNLE would significantly increase the DSP complexity. For example, an *N*-tap FFE would require *N* multiplication and *N*-1 addition operations to equalize one PAM symbol. On the other hand, a truncated VNLE with *N* linear taps and *M* nonlinear taps with beatings of up to *k* neighbors would require $\:N+2\left[Mk-\frac{k\left(k-1\right)}{2}\right]$ multiplications and $\:N+Mk-\frac{k\left(k-1\right)}{2}-1$ additions for the equalization of each PAM symbol. The plot denotes the BER performances of 56.25 GBaud PAM-4, 37.5 GBaud PAM-8, and 42 GBaud PS-PAM-8 signals with square, circle, and star pentagon markers, respectively. For the PAM-4 and PS-PAM-8 signals, we can see that the influence of VNLE is minimal, as it does not provide any substantial gain compared with an FFE. It indicates that when the SOA operates in the linear regime, the bandwidth limitation of the system and ASE noise are the major sources of impairments in system performance. On the contrary, the performance of PAM-8 is still dictated by the residual SOA nonlinearity, as the VNLE can provide a gain of about 1.2 dB at the HD-LDPC decision limit compared with an FFE.

Finally, in Fig. 7(b), we plot the equalized BER results of ≈ 112.5 Gbit/s PAM-4, PAM-8, and PS-PAM-8 signals after 50 km SMF transmission for the application in long-reach PONs. Unlike the BtB transmission case, an improved BER performance can be noticed with a VNLE for both PAM-8 and PS-PAM-8 signals. This is potentially due to the escalated nonlinearities caused by high launch power into the fiber and interactions between the SOA chirp and fiber dispersion that are more detrimental to the PAM-8 and PS-PAM-8 signals. After 50 km, the PAM-8 signal can hardly reach the HD-LDPC level BER performance at an ROP of 0 dBm. Nevertheless, with a VNLE, the PS-PAM-8 can achieve the HD-LDPC BER limit of 1 × 10^− 2^ at an ROP of -5.5 dBm, improving the receiver sensitivity by about 1.2 dB compared to an FFE. Moreover, the PAM-4 signal can reach the same performance level at an ROP of about − 7.05 dBm with an FFE or a VNLE. Even though PAM-4 provides the best BER performance, the reduced symbol period imposes requirements for higher system bandwidth, lower jitter, and low-latency data processing that could potentially increase the implementation cost and complexity. Considering the SOA gain of about 17.3 dB for an input power and bias current of -10 dBm and 125 mA, respectively, signal launch power of 8.5 dBm, and HD-LDPC decision limit of 1 × 10^− 2^, optical power budgets of 32.85 dB and 31.3 dB can be achieved for the PAM-4 and PS-PAM-8 signals, respectively, supporting the class N2 power budget requirements of NG-PON2^33^. When equalized with an FFE, the PS-PAM-8 can still guarantee a class N1 compatible power budget of 29 dB^33^. As mentioned, the total power budget of the LR-PON after 50 km transmission is obtained by adding the feeder and access budgets. In Figs. 7(c)-7(e), the histograms of the recovered PAM-4, PAM-8, and PS-PAM-8 symbols with a VNLE are shown at an ROP of 0 dBm, respectively. From the PAM-4 and PS-PAM-8 histograms, we can see that the probability density function (PDF) of each symbol level is distinct and significantly less non-overlapping compared with the traditional PAM-8. It indicates that the PS can offer robustness against the gain saturation by reducing the probability of occurrence of symbols with higher and lower amplitudes with respect to the inward symbols, resulting in clearer histogram opening with considerably less inter-symbol interference (ISI) after equalization compared with the traditional PAM-8 signals.

## Conclusions

In conclusion, we have experimentally demonstrated single-lane 112.5 Gbit/s PAM-4, PAM-8, and PS-PAM-8 downstream transmissions over 50 km for the application in TDM LR-PONs. Using high launch power and SOA amplification in the RN, we have achieved optical loss budgets of 32.85 dB and 31.3 dB for PAM-4 and PS-PAM-8 signals, respectively, after equalizations using a T-spaced truncated VNLE, satisfying the class N2 power budget requirements of NG-PON2. When equalized with a T-spaced FFE, the PS-PAM-8 modulated LR-PON could still guarantee class N1 power budget requirements after 50 km transmissions. Even though the truncated VNLE shows resilience against SOA nonlinear distortions, more complex and advanced DSPs could enhance the performance.

A typical TDM-PON utilizes a shared feeder fiber for upstream/downstream transmissions in which all the ONUs are connected to the RN via various lengths of distribution fibers to simultaneously transmit/receive data to/from the OLT. This configuration makes the upstream traffic arrival bursty, even though the PON downstream traffic is continuous, as presented in this paper. However, the BER results indicate that the proposed SOA-amplified LR-PON can be operated over a wide dynamic range, making it suitable for the upstream burst-mode operation with a dedicated SOA (one for upstream and one for downstream) in the RN in the presence of nonlinear patterning effects. As discussed, depending on the PON design, the upstream operation can also be realized with a shared SOA in the RN, which shifts the main SOA-induced nonlinear distortions from patterning effects to XGM induced by the upstream(downstream) signals on the downstream(upstream) signals. It is important to quantify the performance of an SOA-amplified bidirectional LR-PON under various SOA configurations with simultaneous bursty upstream and continuous-mode downstream data, which will be an important subject for our future studies. However, the key results presented in this paper related to high capacity and long reach downstream transmission are important for the development of next-generation 100G LR-PONs.

## Data Availability

The datasets generated and or/analyzed during the current study are available from the corresponding author upon reasonable request.
